# From cellular senescence to age-associated diseases: the miRNA connection

**DOI:** 10.1186/2046-2395-1-10

**Published:** 2012-12-03

**Authors:** Elisabeth Schraml, Johannes Grillari

**Affiliations:** 1 Department of Biotechnology, BOKU VIBT University of Natural Resources and Life Sciences, Vienna, Austria; 2 Evercyte GmbH, Muthgasse 18, Vienna, 1190, Austria

**Keywords:** Cellular senescence, Aging, MicroRNA, Non-coding RNA, Age-related diseases, Vascular aging, Osteoporosis, Diabetes mellitus, Kidney disease, Cataract, Sarcopenia

## Abstract

Cellular senescence has evolved from an *in-vitro* model system to study aging *in vitro* to a multifaceted phenomenon of *in-vivo* importance as senescent cells *in vivo* have been identified and their removal delays the onset of age-associated diseases in a mouse model system. From the large emerging class of non-coding RNAs, miRNAs have only recently been functionally implied in the regulatory networks that are modified during the aging process. Here we summarize examples of similarities between the differential expression of miRNAs during senescence and age-associated diseases and suggest that these similarities might emphasize the importance of senescence for the pathogenesis of age-associated diseases. Understanding such a connection on the level of miRNAs might offer valuable opportunities for designing novel diagnostic and therapeutic strategies.

## Review

### Introduction

During aging the incidence of acute and chronic conditions such as neurological disorders, diabetes, degenerative arthritis, and even cancer rises within individuals, so that aging has been termed the substrate on which age-associated diseases grow. Still, the molecular pathways underlying aging are not well understood as large individual heterogeneity of the biological aging process is observed. These interindividual differences are proposed to derive from accumulation of stochastic damage that is counteracted by genetically encoded and environmentally regulated repair systems. At the level of molecules repair works by enzymatic systems while on the cellular level it works by replication and differentiation to maintain tissue homeostasis. However, the replicative potential of somatic and adult stem cells is limited by cellular senescence and recent evidence shows that counteracting senescence or removing senescent cells delays the onset of age-associated pathologies. Here we summarize the current knowledge on how miRNAs might be connecting senescence and age-associated diseases and how such knowledge might be used in the context of biomedical research and medicine.

### Cellular senescence

Replicative senescence was discovered almost 50 years ago when Hayflick observed that normal human cells in culture do have a limited replicative potential [1]. The counting mechanism of the amount of replications was found to be telomere shortening due to the end replication problem [2]. After reaching the replicative limit also termed Hayflick limit, cells enter an irreversible growth arrest that is triggered by critically short, unprotected telomeres that induce a DNA damage like signal [3]. This cell cycle arrest is executed by either of the two important cell cycle inhibitors, p21 or p16, and has so far not been reversible by any known combination of growth factors [4].

### Triggers of cellular senescence

**Figure 1:**
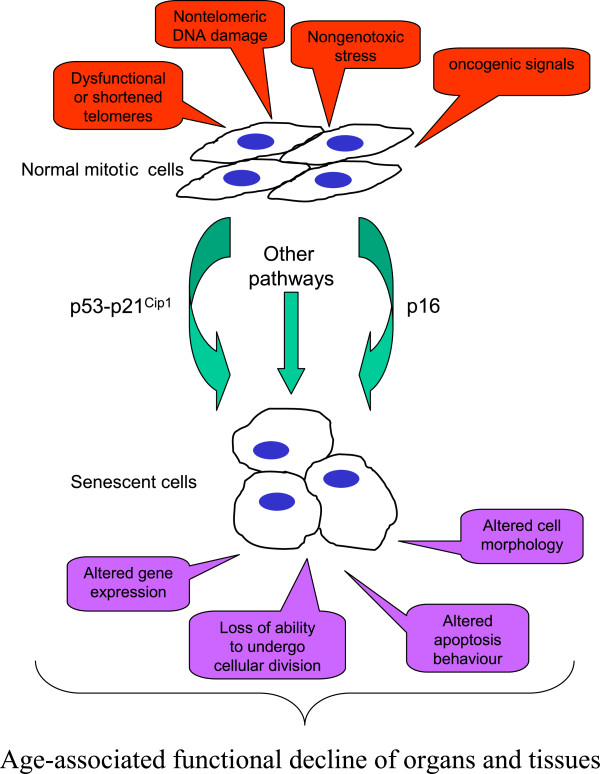
**Age-associated functional decline of organs and tissues**.

By now, several other triggers to a replicative senescence-like irreversible growth arrest have been observed (Figure 1), leading to a the broader term ‘cellular senescence’ that includes: (1) replicative senescence; (2) senescence that is induced by various physico-chemical stressors that induce DNA damage and chromatin disruption, such as, for example, oxidative stress leading to the term stress-induced premature senescence (SIPS); as well as (3) hyperoncogenic signaling-induced senescence, for example by constitutively active HRAS [5,6].

The senescent cell phenotype is characterized by a combination of changes in cell morphology, behavior, structure, and functions. This includes alteration in gene expression [7], protein secretion [8], and inducibility of apoptosis, which increases in senescent fibroblasts [9] and decreases in endothelial cells [10].

#### Cellular senescence in vivo

By now, the presence and age-related accumulation of senescent cells *in vivo* has become well accepted [7,11,12] in various tissues like skin [13], liver [14], kidney [15-17], vasculature [18,19], as well as astrocytes in the cortex of the brain [20,21]. Astrocyte senescence as a component of Alzheimer’s disease.

But is such an accumulation ‘good’ or ‘bad’ for the organism? There seems to no easy answer to this question considering the different faces of senescence [22].

Beneficial functions of senescence include limitation of the extent of fibrosis following liver damage [14]. In addition, senescence has also been well accepted by now as a tumor suppressor mechanism, even *in vivo*. As senescent cells never re-enter the cell cycle, senescence is considered to prevent malignant transformation of potentially mutated cells.

However, some senescent cells also persist within tissues and are not eliminated by apoptosis or the immune system, such that their altered functional profile might alter tissue microenvironments in ways that can promote both cancer and aging phenotypes [22-24]. Especially in regard to age-associated diseases like atherosclerosis [18,19,25] or kidney diseases [26], increasing amounts of senescent cells have been found to at least correlate as will be outlined in more detail below. Causality beyond correlation, however, is supported by the fact that removal of senescent, p16 positive cells in mice delays the onset of at least three prominent age-associated diseases, cataract, sarcopenia, and loss of adipose tissue, even if the model system of BubR1 knock-out mice seems artificial due to its premature aging phenotype [27,28]. Similarly, inducible onset of telomerase reverses age-related functional decline in a third generation telomerase knock-out mouse [29-31] and a gene therapy using hTERT in old mice delays aging and prolongs the life span [32].

Thus, the detrimentally altered functionality of senescent cells might lead to a vicious circle accelerating senescence and/or loss of cells within tissues, resulting in the age-associated decline of body functions and the rise in age-associated diseases. Such altered functionalities are clearly caused by changes in the gene expression pattern of senescent cells, which includes non-coding RNAs and particularly miRNAs.

### MicroRNAs: basics of biogenesis, function, and turnover

MiRNAs comprise a large family of approximately 21-nucleotide-long non-coding RNAs that have emerged as key post-transcriptional regulators of gene expression and have revolutionized our comprehension of the post-transcriptional regulation of gene expression. The first miRNA, lin-4, was discovered by Ambros’s group less than 20 years ago [33]. Since then, the field of small non-coding RNAs has exploded, so that today we are close to developing miRNAs as clinical tools in diagnostics and therapeutic strategies.

**Figure 2:**
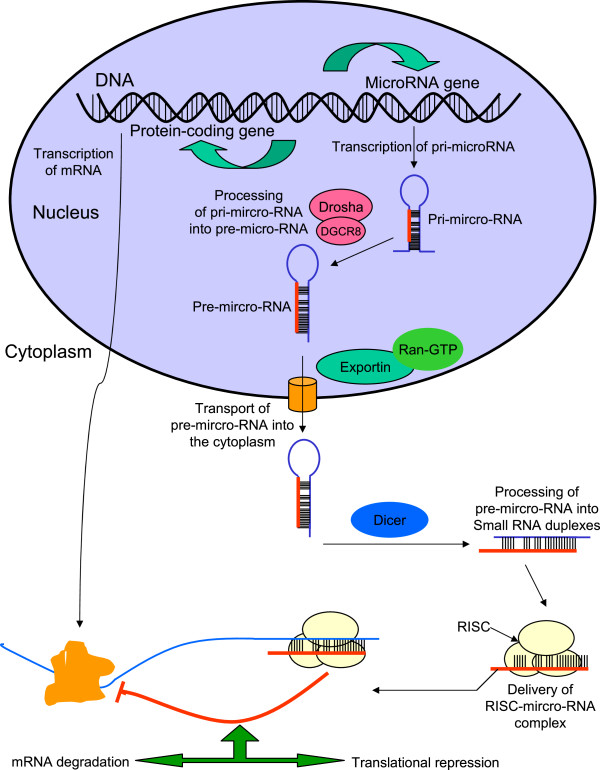
**Overview on miRNA biogenesis and translation repression**.

The biogenesis of miRNAs (Figure 2) involves processing from precursor molecules (pri-miRNAs), which are either transcribed by RNA polymerase II as independent genes or can be derived from introns after splicing [34]. The pri-miRNAs are processed by Drosha to pre-miRNAs, exported to the cytoplasm where Dicer cleaves them to the mature approximately 20-bp miRNA 5p/miRNA 3p duplexes. One strand of this duplex is then incorporated into the miRNA-inducing silencing complex (miRISC) [35].

Silencing of target mRNAs depends on recognition by base-pair mediated binding. This binding is based on a ‘seed’ region consisting of nucleotides 2 to 8 of the miRNA only. This seed can be supported by 3^′^ base-pairing after a short bulge of non-complementarity in the ‘canonical’ binding model. In addition, a shorter seed of down to four nucleotides at the 5^′^ end is still able to silence targets if 3^′^ compensatory complementary supports miRNA-mRNA binding [36]. Due to this ‘loose’ specificity, one miRNA is able to regulate up to 100 mRNA targets and therefore seems to orchestrate a large variety of cellular processes similar to transcription factors [37,38]. While most miRNAs have been reported to bind to the 3^′^ end of their mRNA targets, also 5^′^ end have been identified as miRNA binding sites [39] and binding within the coding sequence has been found [40].

The variability of miRNA/mRNA targeting, however, also includes a ‘miRNA-escape mechamism’ on the side of mRNAs. Alternative polyadenylation has been shown to generate mRNAs that lack the seed regions and thus can evade miRNA-mediated regulation in stem cells [41,42], in quiescent versus proliferating T cells [43,44], but also in cancer cells, where in consequence shorter 30-UTRs arising from alternative cleavage and polyadenylation activate oncogenes [45].

Finally, also miRNA half-life is regulated. It was shown, that miRNAs are subject to degradation by the 5^′^ → 3^′^ exoribonuclease XRN-2 both *in vitro* and *in vivo*. *In vitro*, this process involved miRNA release from AGO, followed by degradation by XRN-2, and both release and degradation were prevented when mRNA was present that had binding sites for the miRNA [46]. *In vivo*, this so-called target mediated miRNA protection (TMMP) acts in opposition to miRNA degradation mediated by XRN-1 and XRN-2 [47].

Summarized, miRNAs are emerging as orchestrators of cell behavior, conferring robustness and balance to biological regulatory loops in many basic biological processes and diseases like cancer. In addition, some functions of miRNAs in controlling aging processes have been uncovered recently as are summarized below: miRNAs regulate lifespan in the nematode Caenorhabditis elegans [48,49], various miRNAs are regulated during mammalian aging in mouse or human tissues [50,51], and, especially, miRNAs have been implicated in governing senescence in a variety of human cells [52-55].

### MiRNAs and cellular senescence

The identification of miRNAs that contribute to induction and maintenance of senescence might also reveal how cellular functions change to allow or even promote induction of age-associated diseases. The general importance of miRNA biogenesis on senescence has been established by the finding that dicer knock-out induces senescence [56].

During the last few years, several studies have then identified differentially transcribed miRNAs during cellular senescence in various cell types and different senescence inducing conditions including fibroblasts [57-63], keratinocytes [64,65], endothelial cells [51,66,67], renal cells, [51,68], T-cells [51], human mesenchymal stem cells of different origins [69,70], UVB-induced senescence of fibroblasts (Greussing *et al*., in revision), mouse embryonic fibroblasts [71,72], trabecular meshwork cells [73], and oncogene-induced senescence in human mammary epithelial cells [74]. Most of these miRNAs are still functionally uncharacterized and might be regulated as a consequence of senescence, and thus contribute to the cellular phenotype of senescence. However, some miRNAs are by now clearly involved in the regulation of senescence.

With regard to cell cycle regulation, we outline here only a few examples of miRNAs that are involved in regulating the senescent phenotype, in particular the let-7 family of miRNAs which inhibits KRAS, HMGA2, and c-MYC. In addition, let-7 is involved in aging of the testis stem cells in Drosophila melanogaster [75]. Similarly, miR15a/16-1 cluster and the miR-17-92 cluster are potent regulators of cell cycle progression by targeting CDK6, CARD10, and CDC27 as well as the CDK inhibitor family members p21, p27, and p57 as reviewed recently [76]. Members of the miR-17-92 cluster, the first identified ‘oncomiR’, has also been found as a commonly downregulated microRNA cluster in human replicative [51,77], and stress-induced senescence [73], as well as organismal aging models. Indeed, inhibition of members of this cluster induces a senescent-like state in human fibroblasts [78], while its upregulation inhibits oncogene-induced senescence [79]. This indicates that this cluster is one additional important player not only in the complex regulatory network of cell cycle and tumorigenesis, but also in aging, emphasizing that these processes are intricately interwoven [52].

With regard to altered functionality of senescent cells, such as, for example, the secretion of cytokines, it is of note that miR-146, which is upregulated in senescent fibroblasts [59] as well as in endothelial cells, is an inhibitor of IL-6 and thus might contribute to the protein secretion alterations observed in senescent cells [59] termed the senescence-associated secretory phenotype (SASP). In addition to such a pro-inflammatory status, members of the miR-200 family that is causally regulating epithelial to mesenchymal transition (EMT), which is an important process in fibrotic as well as metastatic events, has been found as differentially regulated in metformin stress-induced senescence of human fibroblasts [80] as well as in oxidative stress induced senescence of endothelial cells [81]. Finally, miR-24 that is regulated in T cell senescence is also involved in reducing the DNA damage resistance of these cells and thus might contribute to depletion of CD28(−) CD8(+) T cells (Brunner, 2012 #10142).

### Replicative senescence, miRNAs, and age-associated diseases

#### Aging is the substrate on which age-associated diseases are growing

The processes underlying normal aging include accumulation of damage and lack of repair on molecular, cellular, and tissue level ultimately leading to the progressive decline of body functions. Such a decline seems to be an initial event in the pathogenesis of several diseases. Those pathologies that show rapidly increased incidence with higher age and that have advanced age as a single important risk factor are categorized as age-associated diseases. We here rely on a classification of age-associated diseases recently compiled into a comprehensive list by George Martin and colleagues [82] and will here put emphasis on those pathologies that have been connected to cellular senescence (Additional file 1: Table S1). In addition we also summarized all of these most common diseases of the elderly (Table 1). Although many types of cancer can definitely be classified as age-associated diseases, this is not the focus of this review and we kindly recommend some of the very good reviews in the field of cancer and miRNAs [83-85].

**Table 1:** MiRNAs associated with the most common age-related diseases

**Disease**	**miRNA**	**Disease**	**miRNA**
Atherosclerosis,	miR-21	Kidney disease	miR-200a [86]
Ateriosclerosis	miR-210		miR-200b
Ischemic heart disease	miR-34a		miR-141
	miR146a/b [87]		miR-429
	miR-126 [88]		miR-205
	miR-181 [89]		miR-192
	miR-17-19 [90]		miR-194 [91]
	miR-150 [92]		miR-204
	miR217 [93]		miR-215
	miR-143 [94]		miR-216
	miR-145 [95]	Osteoarthritis	miR-133 [96]
	miR-125b [97]	Osteomalacia	miR-135
Diabetes mellitus,	miR-375 [98]	Osteoporosis	miR-29 [99]
type2	miR-130a [100]		miR-233 [101]
	miR-200 [100]	Cataracts	let-7 [102]
	miR-124a [103]		miR-184 [104]
	miR-410 [100]		miR-204 [105]
	miR-122 [106]	Sarcopenia	miR-489 [107]
Kidney disease	miR-17 [108]		miR-1 [109]
	miR-29 [110]		miR-206 [111]
	miR-33 [106]		

#### Senescence, miRNAs, and cardiovascular diseases

Cardiovascular diseases (CWD) (such as atherosclerosis, diabetes, and hypertension) are the primary cause of death and disability in the Western world. These diseases have long been considered to be age-related in terms of their onset and progression [112]. Vascular aging is associated with endothelial dysfunctions [113-115], arterial stiffening and remodeling [116], impaired angiogenesis [117], defects in vascular repair [118], and with an increasing prevalence of atherosclerosis [114,119].

A common characteristic of atherosclerosis is neointimal formation, that is alteration of endothelial cell (EC) physiology and hypoplasia of vascular smooth muscle cells (VSMC), which produce a multi-layered compartment internally to the tunica media of the arterial wall, including a gradual narrowing of the vessels lumen which may lead to thrombus formation and vessel occlusion [120].

The reasons for these associations are still unclear, but it is plausible that organismal aging and vascular disease may share common cellular mechanisms. Especially in regard to cellular senescence *in vivo*, senescent ECs as well as VSMCs have been connected to atherosclerosis [18,19]. The association between vascular pathology and modification of gene expression gives a reasonable expectation that miRNAs may have a central role in the pathogenesis of vessel diseases (Figure 2).

#### Endothelial cell senescence and miRNAs

**Figure 3:**
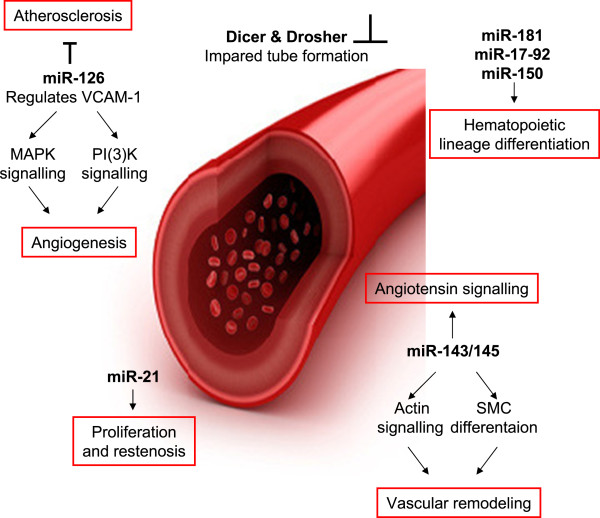
**MiRNAs associated with age associated vascular diseases**.

The importance of miRNAs in endothelial physiology (Figure 3) was revealed for the first time through the *in-vitro* disruption in human ECs of Dicer and Drosha [121-123]. ECs lacking either of these two enzymes showed an impaired ability to form tube structures on matrigel [123]. The generation of an endothelial-specific Dicer knock-out mouse model provided direct evidence that miRNAs are fundamental for the correct vessel development in adulthood in response to angiogenic stimuli [121]. In addition, miRNAs in the serum have been proposed as diagnostic markers for vascular diseases [124-126].

In atherosclerosis, an inflammatory response plays a central role in disease progression. In order to maintain the influx of leucocytes to the lesion areas, ECs increase expression of vascular cell adhesion molecules, such as VCAM-1. One of the most abundant miRNAs in endothelial cells, miR-126, directly represses VCAM-1 expression, thus playing an important role in leucocyte recruitment on the endothelial side [88]. Indeed, miR-126 is downregulated in human aortic endothelial cells [67], and circulating levels of VCAM-1 are increased in elderly human subjects [127]*in vivo*, in stress-induced senescent HUVECs *in vitro*[128], as well as on the surface of endothelial cells in rats *in vivo* and in senescent rat ECs *in vitro*[129]. Therefore, this might contribute to a pro-inflammatory status that allows for disease progression and might explain why upregulated VCAM-1 is suspected to be a causal factor in the pathogenesis of atherosclerosis [130] and is downregulated by a SCM-298, a substance that reduces formation of atherosclerotic plaques in rabbits [131].

Similarly, miR-217 upregulation in human atherosclerotic plaques was observed [66]. *In-vitro* senescent ECs also show higher levels of miR-217 than early passage cells and functionally, miR-217 was able to induce premature EC senescence with SirT1 as target mRNA [66]. Moreover it was shown, that SirT1 acts in complex with FOXO3, a factor involved in modulating longevity in several model systems also regulates senescence in human cell cultures [93]. Of note, a prominent miRNA highly expressed in senescent cells and inducing cellular senescence, miR-34, also converges on SirT1 as a target. Since high levels of SirT1 have been found protective against atherosclerosis by several different studies as reviewed [132], high levels of SirT1 targeting miRNAs as observed in endothelial senescence might indeed contribute to disease progression.

#### Vascular smooth muscle cell senescence and miRNAs

Not only endothelial cells, but also vascular smooth muscle cells (VSMCs) play a major role during events of arterial remodeling and atherosclerosis development. Indeed, miR-21 has been found to be deregulated in EC [67] and fibroblast senescence, as a regulator of neointima lesion formation [133]. Downregulation of aberrantly expressed miR-21 decreased neointima formation in rat carotid artery after angioplasty which classifies miR-21 as a potential therapeutic target [133]. Furthermore, miR-143 and miR145 were reported to be downregulated in VSMCs during neointimal formation in rats [133] and that dysregulation of miR-143 and miR-145 genes is causally involved in the aberrant VSMC plasticity encountered during vascular disease [95]. Indeed, miR-143 is also regulated during senescence, although it has been reported only in fibroblasts so far [134].

#### Diabetes mellitus, type 2

Type 2 diabetes mellitus (T2D) has reached epidemic proportions worldwide [135]. It is estimated that the current 150 million to 220 million people with diabetes will rise up to 300 million in 2025 [136]. T2D is a progressive metabolic disorder characterized by reduced insulin sensitivity, insulin resistance in tissues such as skeletal muscle, liver and adipose tissue, combined with pancreatic β-cell dysfunction, resulting in systemic hyperglycemia [137]. Improper treatment of T2D can lead to severe complications such as heart disease, stroke, kidney failure, blindness, and nerve damage [138].

Cell senescence has recently been postulated as an important cause/consequence of type 2 diabetes and its complications [139].

Circulating miRNAs have also been identified here as potential diagnostic tools [140]. Interestingly, one of the robustly down-regulated miRNAs in this study is miR-126 that is also downregulated in senescent endothelial cells (see above).

Senescence has also been implicated in insulin secretion, since hTERC knock-out mice in the third generation are defective in insulin secretion and glucose metabolism [141]. Remarkably, hTERT gene therapy in old mice restored the age-dependent loss of insulin sensitivity [32]. Similarly, several miRNAs have been implicated in insulin secretion like miR-375, which is one of the most abundant miRNAs in pancreatic islets and beta cells and inhibits insulin secretion via myotrophin (Mtpn) [98]. Mtpn controls release of the neurotransmitter catecholamine [142], that in turn triggers insulin release [98]. In terms of cellular senescence, miR-375 has only been implicated in a chemotherapeutically-induced senescence of the tumor cell line K562.

More recently, miR-130a, miR-200, and miR-410 also were described to be involved in the regulation of insulin secretion [100] and at least members of the miR-200 family are known to contribute to senescence [81].

MiRNAs are not only involved in regulating insulin secretion, but also control insulin signaling in insulin target tissues. In Goto-Kakizaki (GK) rats, which are used as a non-obese model of T2D, members of the miR-29 family are elevated in muscle, fat, and liver, the most important insulin-responsive tissues [143]. This might be causally related to loss of insulin responsiveness, since overexpression of miR-29 *in vitro* in 3 T3-L1 adipocytes also inhibits insulin and glucose responses. This effect might be due to silencing insulin-induced gene 1 (Insig 1) and caveolin 2 (Cav2) [143], two key insulin-responsive proteins. It is of note that miR-29 is also upregulated during cellular senescence [144].

**Figure 4:**
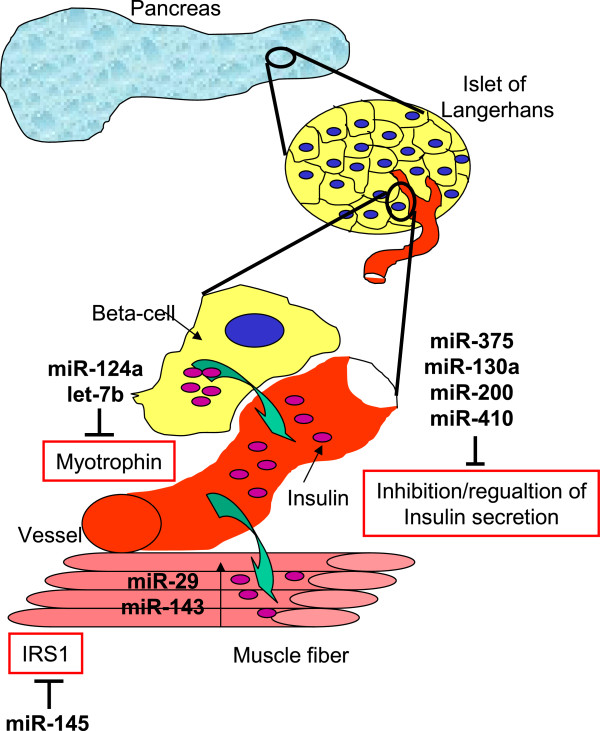
**MiRNAs associated with Type 2 Diabetes mellitus**.

It can be expected that insulin signaling is also directly regulated by miRNAs. Major players in this pathway are insulin receptor substrate (IRS) proteins. Indeed, miR-145 is established as regulator of IRS1 [145] (Figure 4), however, for IRS2, the central player in the development of T2D and its associated complications, no experimentally confirmed target has been identified so far.

#### Kidney diseases

Almost all types of kidney diseases are more common in the elderly having higher risk and incidence of both acute kidney injury (AKI) and end-stage renal disease (ESRD). The precursor state of ESRD, namely generic chronic kidney disease (CKD), is also much more common in the elderly [146]. Furthermore, fibrotic events also diminish kidney functionality. This loss of functionality again is correlated with increase of senescent cells in the kidney [15,26,43,147]. Furthermore, high amounts of senescent cells in kidneys for transplantation are correlated with low transplantation success [16,17], supporting the idea that senescent cells are ‘bad citizens’ and ‘bad neighbors’ in the kidney of the elderly. MicroRNAs have already been found to be involved in senescence of different kidney cells. In rat mesangial cells, miR-335 as well as miR-34c promote senescence by suppressing antioxidative defense proteins [68]. Loss of miR-335 expression has been found in patients of renal cell carcinoma, which might be in keeping with the idea that miR-335 can act as a tumor suppressor by inducing senescence [148].

In addition, senescent renal proximal tubular epithelial cells have high levels of miRNAs of the miR-200 family including miR-205 [51]. It seems that therefore, senescence of RPTECs and fibrosis might be linked [149]. Indeed, EMT seems to be regulated in renal fibrinogenesis by this family, and miR-200 can ameliorate this condition. It was shown, that five members of the miR-200 family (miR-200a/b/c, miR-141, and miR-429) and miR-205 are specifically downregulated in MDCK cells undergoing EMT [150]. Moreover, miR-200b ameliorates tubulointerstitial fibrosis in obstructed kidneys and thereby might constitute a novel therapeutic targets in kidney disease [151]. Subsequently, the function of the miR-200 family in regulating ZEB1 and ZEB2 and in modulating EMT in a number of different cell types has been confirmed [152-155].

**Figure 5:**
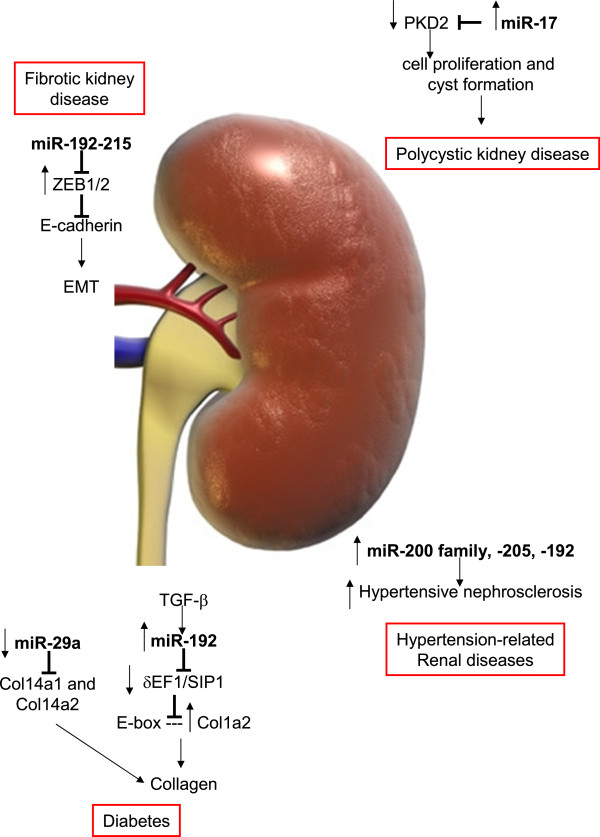
**MiRNAs in age associated disease of the kidney**.

Similarly important for regulating EMT in the kidney are miRNA-192/215 [156], two miRNAs that are specifically high in kidney tissue [91] (Figure 5). miR-192, in particular, also plays a role in diabetic nephropathy [157], as its loss correlates with tubulointerstitial fibrosis and reduction in eGFR in renal biopsies from patients with established diabetic nephropathy [158].

Finally, miR-29 represses the expression of collagen I and IV at both the mRNA and protein level [110] and is downregulated in senescent RPTECs [51], thus it might contribute to more fibrinogenic material in the aged kidney.

In keeping with the above, in 34 consecutive patients with biopsy-proven hypertensive nephrosclerosis, a progressive disease that results from sclerosis of the small blood vessels in the kidney and is most commonly associated with hypertension or diabetes, intrarenal expression of miR-200a, miR-200b, miR-141, miR-429, miR-205, and miR-192 were increased, and the degree of upregulation correlated with disease severity [159]. Taken together, both cellular senescence as well as miRNAs regulated in cellular senescence have been found to negatively impact on kidney functionality. We therefore suggest that the link between senescence, miRNAs, and kidney disease might not only be correlative, but causal in the aging kidney.

#### Osteoporosis

The skeleton is continuously remodeled throughout the lifetime of an individual in a dynamic process of bone resorption and bone formation, to replace damaged bone or to respond to metabolic needs [160]. This bone turnover is mediated by the delicate balance of osteoblast and osteoclast numbers and activities. Osteoclasts resorb bone, whereas ostoeblasts synthesize new bone [161]. Dysregulation of either one of these cell types therefore results in an imbalance of bone turnover and pathological consequences, including osteoporosis in case of prevalent bone resorption, resulting in excessive skeletal fragility leading to frailty and a high risk of low-trauma fractures.

Hints for the importance of cellular senescence in the development of osteoporosis come from hTERC knock-out mice [162,163], hTERT gene therapy that delays the onset of osteoporosis in old mice [32] as well from the fact that removal of senescent p16^+^ cells also delays the onset of skeletal deformation in the progeroid BubR1 deficient mouse [27]. The cell types mainly studied with regard to senescence are mesenchymal stem cells that are the progenitors for osteoblasts. It has been shown that the replicative potential of MSCs clearly depends on the age of the donor [164], a fact that is not so clear for fibroblast strains [165].

**Figure 6:**
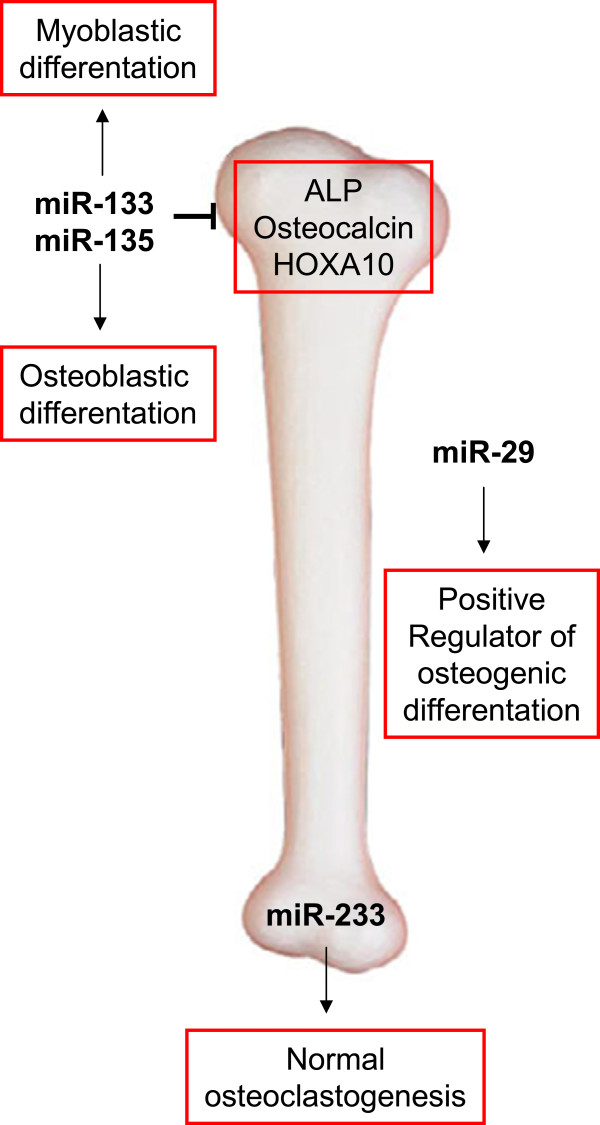
**MiRNAs associated with osteoporosis**.

Although, a clear physiological link between osteoporosis and the loss of replicative potential of cells seems to exist, too few studies have yet addressed miRNAs and MSC senescence. Still, we want to point out some candidate miRNAs that have been found to play a role during the differentiation from MSCs to osteoblasts (Figure 6), among them miR-637 [166], miR-133 and miR-135, the miR-29 family [99,167], and miR-138 [168]. In regard to osteoclasts, only few reports exist and identify miR-233 to reduce formation of osteoclast-like cells in RAW264.7 mouse cells as model system [101,169].

However, so far only miR-2861 has been implicated functionally in osteoporosis, as silencing of it *in vivo* in mice reduced bone formation and bone mass [170]. In addition, mutations in the pre-miR-2861 in two patients result in lack of mature miR-2861, causing primary osteoporosis [170].

#### Cataract

Cataracts are a very common eye pathology with advanced age being one of the most prominent risk factors. Most people above the age of 65 years show some changes in lens structure and most will develop a cataract in time [171]. Recently it was shown that cataract formation was significantly accelerated in *BubR1*^H/H^ mice. However, by removal of senescent cells the onset of cataract formation was significantly delayed, emphasizing the importance of senescence in this regard [27,28] as well as by hTERT gene therapy [32].

Moreover, it was shown that miRNAs play a role in age-related cataracts [102]. Let-7 miRNA, an important regulator of cellular aging and tissue senescence [102], was demonstrated to be positively associated with patient age and a positive correlation was also observed between cataract and higher expression of let-7b miRNA in patients with age-related cataracts [102]. Moreover it was shown that miR-184 and miR-204 play a role in formation of secondary cataracts, formed mostly after eye surgery, or caused by diabetes or steroid use [105].

#### Sarcopenia

Sarcopenia can be defined as the age-related loss of muscle mass, strength, and function, and appears to begin in the fourth decade of life and accelerates after the age of approximately 75 years [109,172]. While many factors contribute to the onset of sarcopenia, one of the main causes is a change in the nature of a small population of muscle stem cells, also called satellite cells. Similar to cataracts, skeletal muscle degeneration was greatly reduced in *BubR1*^H/H^ muscles after removal of senescent, p16 positive cells [27,28]. In addition, senescence of muscle cells and satellite cells seems to be implicated in muscle metabolism and disease [173-175].

So far, however, only a few miRNAs were found to be implicated in satellite cell regulation. MiRNA-489 is highly expressed in quiescent satellite cells and is quickly downregulated during satellite-cell activation [107]. Further analysis revealed that miR-489 functions as a regulator of satellite-cell quiescence, as it post-transcriptionally suppresses the oncogene *Dek*, the protein product of which localizes to the more differentiated daughter cell during asymmetric division of satellite cells and promotes the transient proliferative expansion of myogenic progenitors [107]. Moreover miR-1 and miR-206 can improve human satellite cell differentiation via repressing Pax7, a central player in satellite cell survival, self-renewal, and proliferation [111,176]. No data are yet available which link these miRNAs to cellular senescence. It will be interesting to what extent such a connection might exist, especially in view to the role of the systemic environment on satellite cell function, since in heterochronic parabiosis of young and old mice the proliferation and regenerative capacity of aged satellite cells was ‘rejuvenated’ [177,178].

#### Other age-related diseases

Many more age-associated diseases are known (Additional file 1: Table S1), among them Alzheimer’s disease (AD), Parkinson’s disease, degenerative arthritis, and destructive eye diseases. Except for AD and Parkinson’s disease, to date no reports exist linking miRNAs to these diseases and very recent reviews are available on miRNAs in neurodegenerative diseases [179]. Similarly, we want to refer the reader to recent reviews on miRNAs and cancer [83-85], which is one of the important age-related diseases. In cancer, miRNAs have a potential value as tumor markers and it was shown that deregulation of miRNAs not only results as consequence of cancer progression but also directly promotes tumor initiation and progression in a cause-effect manner.

## Conclusion

As cellular senescence is becoming ever more prominent as a mechanism that can drive aging and promote age-related diseases, one of the questions that is only poorly answered remains: how many senescent cells can be found in the elderly in specific tissues and what are the functional changes that tissue specific cells undergo when senescent, as it is clear that cell types as diverse as fibroblasts and epithelial or endothelial cells also will greatly vary when senescent. The comparison of miRNAs involved in cellular senescence to miRNAs involved in age-associated diseases shows that surprisingly many miRNAs are shared in these *in-vitro* and *in-vivo* situations. While it is clear that these similarities are merely correlative, a more detailed study on causal links might be a good approach to identify novel diagnostic and therapeutic strategies for age-associated diseases. In addition, since miRNAs are only a small part of the emerging non-coding RNA field, other ncRNAs might emerge equally important for the understanding of the aging process and the pathogenesis of age-associated diseases.

## Competing interests

JG is co-founder and CSO of Evercyte Gmbh.

## Authors’ contributions

ES planned and wrote the manuscript. JG planned, designed and wrote the manuscript. All authors read and approved the final manuscript.

## Supplementary Material

Additional file 1 **Table S1.** Summary of age-associated diseases. Click here for file
